# Enabling unassisted solar water splitting by iron oxide and silicon

**DOI:** 10.1038/ncomms8447

**Published:** 2015-06-16

**Authors:** Ji-Wook Jang, Chun Du, Yifan Ye, Yongjing Lin, Xiahui Yao, James Thorne, Erik Liu, Gregory McMahon, Junfa Zhu, Ali Javey, Jinghua Guo, Dunwei Wang

**Affiliations:** 1 Department of Chemistry, Merkert Chemistry Center, Boston College, 2609 Beacon St, Chestnut Hill, Massachusetts 02467, USA; 2 Advanced Light Source, Lawrence Berkeley National Laboratory, Berkeley, California 94720, USA; 3 National Synchrotron Radiation Laboratory, University of Science and Technology of China, Hefei 230029, China; 4 Department of Electrical Engineering and Computer Sciences and the Joint Center for Artificial Synthesis, University of California, Berkeley, California 94720, USA

## Abstract

Photoelectrochemical (PEC) water splitting promises a solution to the problem of large-scale solar energy storage. However, its development has been impeded by the poor performance of photoanodes, particularly in their capability for photovoltage generation. Many examples employing photovoltaic modules to correct the deficiency for unassisted solar water splitting have been reported to-date. Here we show that, by using the prototypical photoanode material of haematite as a study tool, structural disorders on or near the surfaces are important causes of the low photovoltages. We develop a facile re-growth strategy to reduce surface disorders and as a consequence, a turn-on voltage of 0.45 V (versus reversible hydrogen electrode) is achieved. This result permits us to construct a photoelectrochemical device with a haematite photoanode and Si photocathode to split water at an overall efficiency of 0.91%, with NiFeO_x_ and TiO_2_/Pt overlayers, respectively.

## Abstract (web summary)

Water splitting using earth-abundant materials promises a low cost solution to the problem of large scale energy storage. Here, the authors fabricate a haematite and silicon-based high-efficiency water splitting device, which operates without the need for an externally applied bias.

## Graphical abstract

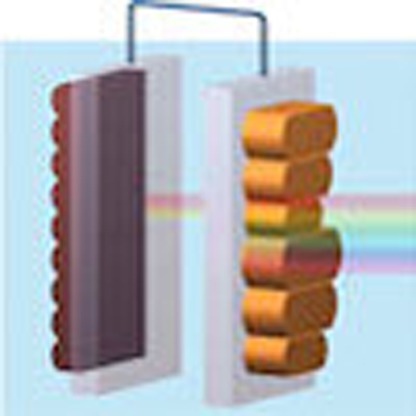

How to carry out solar water splitting (H_2_O → ½ O_2_+reactive protons) efficiently and inexpensively constitutes a great challenge that inspires significant research^1^,^2^,^3^. More efficient and stable than molecular systems and less expensive than simple combinations of photovoltaics and electrolysis, direct photolysis (or photoelectrochemistry, PEC) is particularly appealing^4^. Decades of intense research notwithstanding, complete solar water splitting by PEC without the need for externally applied bias (referred to as unassisted water splitting) remains rare^5^,^6^,^7^,^8^,^9^,^10^,^11^,^12^. Existing examples often include, sometimes entirely powered by, photovoltaic modules^5^,^6^,^7^,^8^,^9^,^10^. The poor performance of photoanodes, especially in their photovoltage generation capabilities, has been a key issue. The challenges involved are probably best exemplified by haematite (α-Fe_2_O_3_), a prototypical photoanode material that has piqued great interest but failed to deliver the expected performance^13^,^14^,^15^,^16^. The most pronounced disadvantage that plagues the promises held by haematite is its low photovoltages, characterized by late turn-ons in its photoelectrochemical behaviours (typically 0.8–1.0 V). For a semiconductor whose reported Fermi level ranges between 0.4 and 0.7 V (versus reversible hydrogen electrode (RHE)), the late turn-on voltages are not fully accounted for and require further understandings.

The light-to-charge conversion of a PEC device is governed by the same photophysics that describes photovoltaics^17^. The intricacy of how the semiconductor/water interface influences both the energetics and kinetics of a photoelectrode, however, makes it difficult to pinpoint the origins of potential loss at the interface^18^,^19^,^20^. This is because both energetics (in the form of photovoltages) and kinetics (in the form of overpotentials) affect the steady-state current/voltage behaviours in similar manners. The complexity has led to a recent debate on the role of surface modifications on haematite by cobalt phosphate-derived amorphous layers^21^,^22^,^23^,^24^. On a parallel system featuring amorphous NiFeO_x_, prepared by a photochemical deposition technique^25^, we showed that significant cathodic shift enabled by the water oxidation catalyst is primarily due to improvement in the interface energetics, but not the water oxidation kinetics, despite the fact that NiFeO_x_ is indeed a good water oxidation catalyst^20^. Inspired by the understandings, here we seek to exploit what can be enabled by surface modifications. It is shown that a facile regrowth strategy readily improves the measured photovoltages on a haematite photoanode. Our results point the origin of the low photovoltages towards short-range structural disorders near the surface of photoanode. A low turn-on voltage of 0.45 (±0.01) V was obtained, enabling unassisted water splitting with amorphous Si as a photocathode^26^ at efficiencies up to 0.9%. Our demonstration represents the first example of unassisted solar water splitting using haematite and Si as the sole light absorbers.

## Results

### Synthesis and PEC performances

**Figure 1:**
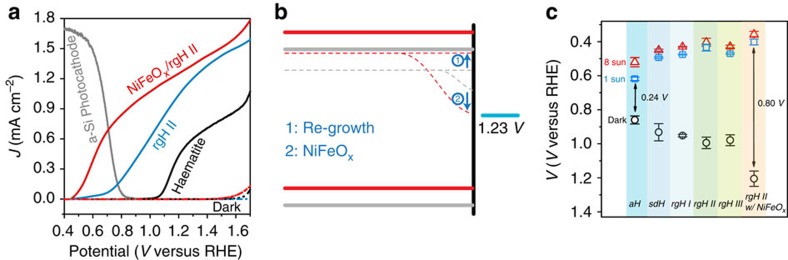
Haematite with radically improved turn-on characteristics. (**a**) Steady-state current density-potential behaviours of various haematite photoelectrodes. The current densities of Si photocathode placed behind the haematite photoanode are shown to illustrate the meeting points. (**b**) Band diagram of unmodified haematite (grey) and NiFeO_x_-decorated haematite after re-growth treatments (red) under flat-band, quasi-equilibrium conditions. The Fermi level shift (denoted as 1) is a direct result of the re-growth treatment. The hole quasi-equilibrium potential shift (denoted as 2) is due to the application of NiFeO_x_. (**c**) Open circuit potential measurements of aH, sdH, rgH I, rgH II, rgH III and NiFeO_x_-decorated rgH II under 8-sun (red, triangle), 1-sun (blue, square) and dark (black, circle) conditions. Throughout this manuscript, sdH refers to solution-derived haematite; rgH I, rgH II, and rgH III denote haematite samples subjected to the regrowth treatments one, two and three times, respectively. Haematite prepared by atomic layer deposition (ALD) and then annealed at 500 °C and 800 °C are labelled aH and aH 800, respectively. NiFeO_x_/rgH II represent rgH II haematite decorated with amorphous NiFeO_x_ catalysts. The error bars were obtained by taking s.d. values of measurements on at least three different samples for each data point.

As previous results suggest defects on or near the surface of haematite may be an important reason for the late turn-on characteristics, we hypothesize that chemistries that alter the surfaces will have a direct impact on the resulting material's PEC behaviours. A solution-based re-growth technique is employed to test the hypothesis, where pre-formed haematite is subjected to acidic solutions under which condition dissolution (of Fe_2_O_3_) and deposition (of FeOOH) occur concurrently (Supplementary Fig. 1). A brief (∼5 min) post-growth annealing at 800 °C converts FeOOH to haematite. The striking effect on the PEC behaviours is evident in Fig. 1a. The turn-on voltages of haematite subjected to the re-growth chemistry at 0.67 (±0.01) V are significantly lower than those of atomic layer deposition (ALD) grown haematite without the re-growth treatments (∼1.05 (±0.02) V). The difference of 0.38 V is well beyond sample variations (Supplementary Fig. 2). It is noted that, although similar turn-on voltages have been obtained by Li and colleagues^27^, and Hamann and Zandi^28^, separately, the overall performance of the photoelectrodes reported by them trail what we report here by a large margin (Supplementary Fig. 3).

### Structural characterization

**Figure 2:**
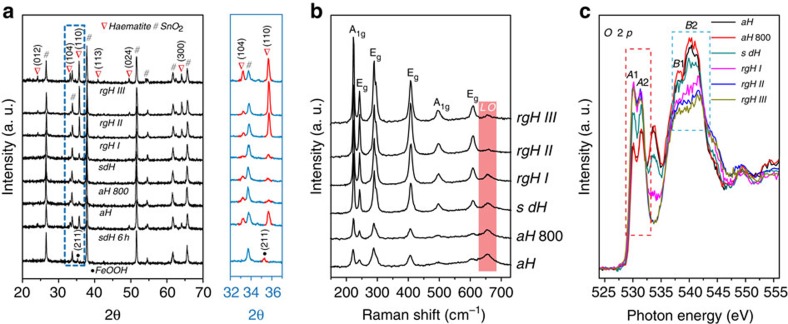
X-ray diffraction, Raman and X-ray absorption analysis. (**a**) X-ray diffraction patterns, (**b**) Raman shift spectra and (**c**) Oxygen K-edge X-ray absorption spectra of aH, aH 800 (ALD-grown haematite annealed at 800 °C in air), sdH, rgH I, rgH II and rgH III. The details of sample IDs can be found in the captions for Fig. 1.

Our focus for the present work is to understand what underpins the strikingly high photovoltages (up to 0.80 V). It is seen from the simplified band diagram (Fig. 1b) that maximized photovoltages require the Fermi level to be as negative as possible relative to the water oxidation potential. Effects within the body (for example, low carrier concentration) and on the surface (for example, partial band edge unpinning) of the photoelectrode may move the Fermi level towards the positive direction. Our first task was to use the re-growth treatments to counteract these negative influences. To observe the effectiveness of the regrowth strategy, we probed the Fermi levels under equilibrium and quasi-equilibrium (that is, open-circuit) conditions. The results are compared in Fig. 1c. The data under intense light report on potentials close to the ‘true' flatband potential (Supplementary Fig. 4), whereas the potentials under 1-sun condition offer a reference point for us to understand the PEC behaviours as shown in Fig. 1a, which were taken under 1-sun illumination. The potentials in dark can be used to inspect whether there are undesired surface Fermi level pinning effects. Examinations of Fig. 1c revealed that ∼0.13 V potential is harvested from the Fermi level shift due to the switch of synthesis methods (*V*_*f*_=0.62 (±0.01) V for ALD haematite, denoted as aH in Fig. 1c; *V*_*f*_=0.49 (±0.01) V for solution derived haematite, denoted as sdH). The difference in *V*_*f*_ is ascribed to the difference in the detailed structures of haematite prepared by various methods, as evidenced by the X-ray diffraction patterns (Fig. 2a).

It is hypothesized that during re-growth, nanoscale structures on the surface of haematite as a result of the FeOOH→Fe_2_O_3_ conversion are dissolved, and the newly grown structures favour the <110> directions. That increased (110)/(104) peak intensity ratios (from 0.64 for aH to 10.1 for rgH II, Supplementary Table 1) correspond to more negative *V*_*f*_ is consistent with the observations made by Peter *et al.*^29^, on haematite synthesized by gas-phase pyrolysis. Consecutive re-growth treatments move *V*_*f*_ towards the negative direction monotonically, reaching 0.44 (±0.02) V after the second re-growth. Interestingly, the corresponding dark equilibrium potentials also change monotonically, towards the more positive position, reaching 0.99 (±0.03) V after the second re-growth. Together, the simple treatments increased the photovoltage (as measured by the difference of *V*_*f*_ in light and in dark) by 27% (from 0.44 to 0.56 V). As the re-growth treatments are only expected to alter the surfaces, changes in Fermi levels described here are ascribed to changes in surface structures (Fig. 1c). Finally, the shift of *V*_*f*_ in dark from 0.99 V to 1.21(±0.04) V upon the application of NiFeO_x_ is consistent with our previous observations^20^. The shift of *V*_*f*_ under illumination from 0.44 V to 0.40 (±0.02) V, although modest, is unexpected. We understand it as a result of improved surfaces by the application of NiFeO_x_. The final photovoltage of 0.80 V is the highest for haematite reported in the literature.

The XRD data further imply that short-range disorder within haematite is reduced by the facile regrowth treatments. The expectation was confirmed by the Raman spectra (Fig. 2b). The peaks at 660 cm^−1^ correspond to the forbidden longitudinal optical (LO) mode. The appearance of the forbidden mode is indicative of symmetry breakdown induced by structural disorders and, hence, scattered LO phonons^30^. Important to our discussions, the peak intensity decreases after each re-growth treatment, supporting that the treatment effectively reduces the disorder. Alternatively, one may argue the repeated annealing of the same substrate at 800 °C (for the conversion of FeOOH→Fe_2_O_3_) is responsible for the changes. Control experiments where sdH was annealed without re-growth treatment yielded an obvious increase of the LO peaks and worsened turn-on characteristics (Supplementary Fig. 5). Furthermore, the LO peak at 660 cm^−1^ by aH was persistent after the 800 °C annealing, accompanied by late turn-on behaviours.

**Figure 3:**
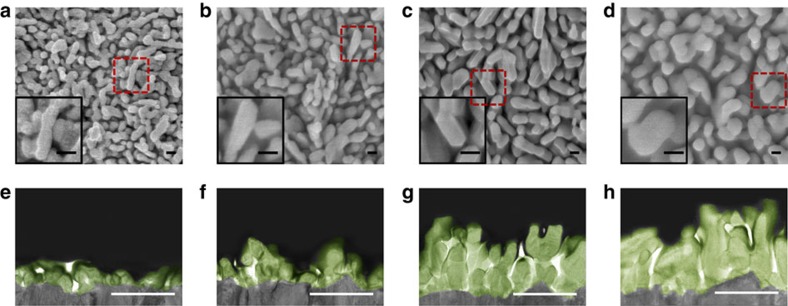
Morphology evolution of haematite as a result of the re-growth treatments. Scanning electron micrographs image of (**a**) sdH, (**b**) rgH I, (**c**) rgH II and (**d**) rgH III; scale bars, 100 nm. Magnified views of selected areas in the main frames are presented in the insets. Transmission electron micrographs of focused ion beam prepared cross-sectional samples of (**e**) sdH, (**f**) rgH I, (**g**) rgH II and (**h**) rgH III, scale bars, 500 nm.

Electron micrographs (Fig. 3) landed us further support on the understanding that the re-growth strategy is effective in re-organizing haematite surfaces. Small (∼70 nm), random structures were most prominent for sdH; the orderliness of the structures was clearly improved after the second regrowth treatment (rgH II). Further regrowth (rgH III), nevertheless, resulted in overgrowth that affects the PEC performance negatively (Supplementary Fig. 2). The PEC behaviours are well corroborated with the changes in the LO peak intensities (Fig. 2b) and increased (104) peak intensities (Fig. 2a).

To further corroborate the structural changes with electronic structures, we next compare the X-ray absorption spectra (XAS) in Fig. 2c. Here, the relative intensities of two prominent peak groups, *A* peaks (*A*1 and *A*2, due to absorptions by hybridized O 2*p* anti-bonding and Fe 3*d* 2t_g_ and e_g_ orbitals) and *B* peaks (*B*1, *B*2, due to absorptions by O 2*p* and Fe 4*s* and 4*p* orbitals) deserve special attention. Less intense *A* peaks and more intense *B* peaks are indicative of greater contribution by Fe-O with reduced oxygen contents (that is, Fe sites of low coordination numbers). They are understood as the origin of structural disorders^31^. Of the samples studied, the *A* peak intensity of sdH is much higher, and the *B* peak intensity of sdH is much lower, than those of aH and aH 800, respectively. The result provides a direct proof of higher degree of structural disorder in aH. Significantly, re-growth treatments markedly increase the *A* peaks and decrease the *B* peaks. Because the regrowth treatments do not completely dissolve the preformed haematite, the consistent changes in XRD, Raman and XAS data strongly support that the structural disorder primarily resides near the surface. Such a feature is advantageous as it allows us to readily alter the degree of the disorder by changing the surfaces. Finally, while our data do not rule out the possibilities that the Fermi level shift as observed in Fig. 1c is a result of doping effect, the carrier concentration needs to be increased by more than three orders of magnitude to fully account for the changes measured, far from the variations of our measured carrier concentrations (Supplementary Fig. 6, Supplementary Table 2 and Supplementary Discussions). We therefore consider the doping effect an insignificant factor in the Fermi level shift.

Taken as a whole, we understand the Fermi level shift between aH and sdH (0.13 V) as a result of structural difference. While aH feature grains without obvious preferences of their orientations, sdH appears to favour growth of (110) planes. The preference has been previously reported^32^ and was understood as the matching lattice distances of FeOOH (221) planes (2.5 Å) and haematite (110) planes (2.5 Å; also see Fig. 2a).

### Overall unassisted water splitting

**Figure 4:**
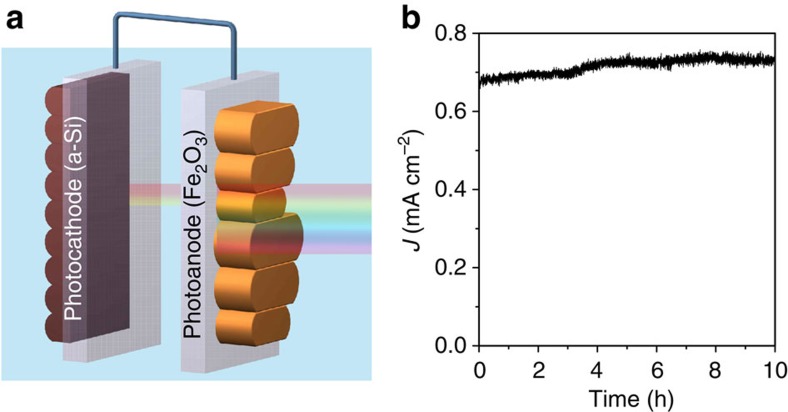
Overall unassisted water splitting. (**a**) Schematics of overall unassisted water splitting by haematite photoanode (right) and amorphous Si photocathode (left) in a tandem configuration. (**b**) Net photocurrent during the first 10 h of operation using NiFeO_x_-modified rgH II with TiO_2_/Pt-loaded amorphous silicon photocathode in 0.5 M phosphate solution (pH 11.8) in a two-electrode, tandem configuration (no external bias).

The turn-on voltage of 0.45 V by haematite is comparable to the turn-on potentials by WO_3_ or BiVO_4_ (refs 9, 10, 33). Because the bandgap of haematite (∼2 eV) is considerably smaller than that latter (2.8 and 2.4 eV, respectively), the room for improvement by haematite is greater. We next demonstrate the first unassisted water splitting by haematite. Our chosen photocathode features TiO_2_/Pt loaded amorphous Si, a recently demonstrated cathode featuring internal p-i-n junctions for high photovoltages^26^. The configuration is shown in Fig. 4a, with the light passing haematite photoanode first. The electrolyte was buffered by phosphate at pH 11.8. The efficiency as measured by the photocurrents, in the absence of any externally applied bias, approached to 0.91% (Supplementary Table 3, Fig. 4b). The quantities of H_2_ and O_2_ as detected by mass spectrometry match the charges measured, proving the Faradaic efficiencies are close to 100% (Supplementary Fig. 7). Stabilities test showed no obvious decay during the first 10 h (Fig. 4b). When the Si photocathode was exposed to illuminations without pre-absorption by haematite photoanode (and hence significantly stronger intensity in the UV and visible region), it decayed 12.7% in the first 10 h (Supplementary Fig. 8). No decay was measured on the haematite photoanode (Supplementary Fig. 9).

## Discussion

When compared with other reported efficiencies involving photovoltaic modules, the 0.91% exhibited by haematite and Si is indeed modest. However, it is the first time a meaningful efficiency ever measured by haematite and Si, which are made of the most abundant elements on Earth. We envision the efficiency can be readily improved by, for instance, increasing photocurrents of haematite (the highest photocurrent of haematite photoanode to-date was 4.32 mA cm^−2^ at 1.23 V versus RHE)^34^ or optimizing light absorption of haematite photoanode (the absorption of the haematite photoanode in this work reduced the photocurrents of Si photocathode by five folds, Supplementary Fig. 10). Once optimized, the reported approach of using Fe, O and Si to split water is amenable to large-scale utilization. More broadly, the facile re-growth strategy may find utilities in other photoelectrodes for maximized photovoltage generation in photoelectrochemical systems.

## Methods

### Haematite synthesis

FeOOH was grown on fluorine-doped tin oxide (FTO) substrates (∼7 Ω sq^−1^, Sigma) in a solution containing 0.15 M iron (III) chloride hexahydrate (FeCl_3_, 97%, Alfa Aesar) and 1 M sodium nitrate (NaNO_3_, 99%, Alfa Aesar). The reaction was carried out at 100 °C for 1 h. After rinsing with DI water, calcination was performed at 800 °C for 5 min to convert FeOOH into haematite. In this paper, solution-derived haematite is denoted as sdH. Haematite after one re-growth treatment (100 °C growth for 1 h, then 800 °C annealing for 5 min) is labelled as rgH I; rgH II and rgH III refer to haematite samples that have been treated by the growth conditions twice and thrice, respectively. To confine the growth haematite only on the front side of the FTO substrate, Kapton tapes were applied to cover the backside during the growth. The tapes were removed before post-growth calcination.

### NiFeO_x_ deposition

Iron (III) 2-ethylhexanoate (50% w/w in mineral spirits, Strem Chemicals) and nickel (II) 2-ethylhexanoate (78% w/w in 2-ethylhexanoic acid, Strem Chemicals) were mixed in hexane solvent to achieve a total concentration of 15% w/w metal complex solution^26^. The solution was further diluted with hexane to one-tenth of its original concentration. Approximately 10 μl cm^−2^ precursor solution was directly drop casted on a nonconductive epoxy-covered haematite electrode-exposed area. After drying in air for 5 min, the electrode was irradiated with a UV light (intensity: ∼21 mW cm^−2^) for 5 min. Afterwards, the electrode was annealed in an oven at 100 °C for 1 h.

### PEC characterizations

All PEC characterizations were carried out using a potentiostat/galvanostat (CH Instruments CHI604C) and the light source was an AM 1.5 solar simulator (100 mW cm^−2^, Newport Oriel 96,000) calibrated by a thermopile optical detector (Newport, Model 818P-010-12). In a typical test cell, haematite/FTO electrode was used as the working electrode, a Pt wire served as the counter electrode, and a Hg/HgO (or a Ag/AgCl) electrode was used as the reference electrode depending on the pH of the electrolyte. The scan rate was 10 mV s^−1^. For measurements under varying lighting conditions (1-sun to 8-sun), the light source was a solar simulator (Newport Oriel, Model 6297NS) equipped with IR water filter whose intensity was adjusted using a thermopile optical detector (Newport, Model 818P-010-12). To measure the open-circuit potentials, each dark/light potential reading was obtained after stabilization for at least 30 min with constant stirring while oxygen gas was bubbled into the electrolyte solution.

The complete water splitting cell was constructed in a phosphate buffered electrolyte (pH 11.8). The a-Si photocathode used in this study was fabricated using the same method as reported previously^17^. A potentialstat and a sourcemeter (Keithley 2,400) were used to evaluate the operation current in the two-electrode system. Product detection of solar water splitting (H_2_ and O_2_) was conducted using a mass spectrometer (MKS V2000P).

### Material characterizations

The samples were characterized by a scanning electron microscope (SEM, JSM6340F), a transmission electron microscope (TEM, JEOL 2010F, 200 kV), a micro-Raman system (XploRa, Horiba) with 532-nm laser excitation, an X-ray absorption spectrometer (a channeltron at beamline-8.0.1 at the Advanced Light Source, Lawrence Berkeley National Laboratory), an X-ray diffractometer (XRD, PANalytical X'Pert with Cu *K*_α_ radiation) and an integrating sphere from SphereOptics (Ocean Optics USB 4,000). For cross-sectional TEM samples, haematite electrodes were milled by a focused ion beam (FIB, JOEL 4,500 multibeam system) microscope. A layer of W film was first deposited on top of the samples before milling to minimize ion beam damage.

## Additional information

**How to cite this article:** Jang, J.-W. *et al.* Enabling unassisted solar water splitting by iron oxide and silicon. *Nat. Commun.* 6:7447 doi: 10.1038/ncomms8447 (2015).

## Supplementary Material

Supplementary Information Supplementary Figures 1-10, Supplementary Tables 1-3, Supplementary Discussion and Supplementary References
