# Supplementary material for: Enabling unassisted solar water splitting by iron oxide and silicon
Source: Nat Commun. 2015 Jun 16;6:7447. doi: 10.1038/ncomms8447 (PMC4490416; doi:10.1038/ncomms8447)
Supplement: Supplementary Information — Supplementary Figures 1-10, Supplementary Tables 1-3, Supplementary Discussion and Supplementary References [file ncomms8447-s1.pdf]

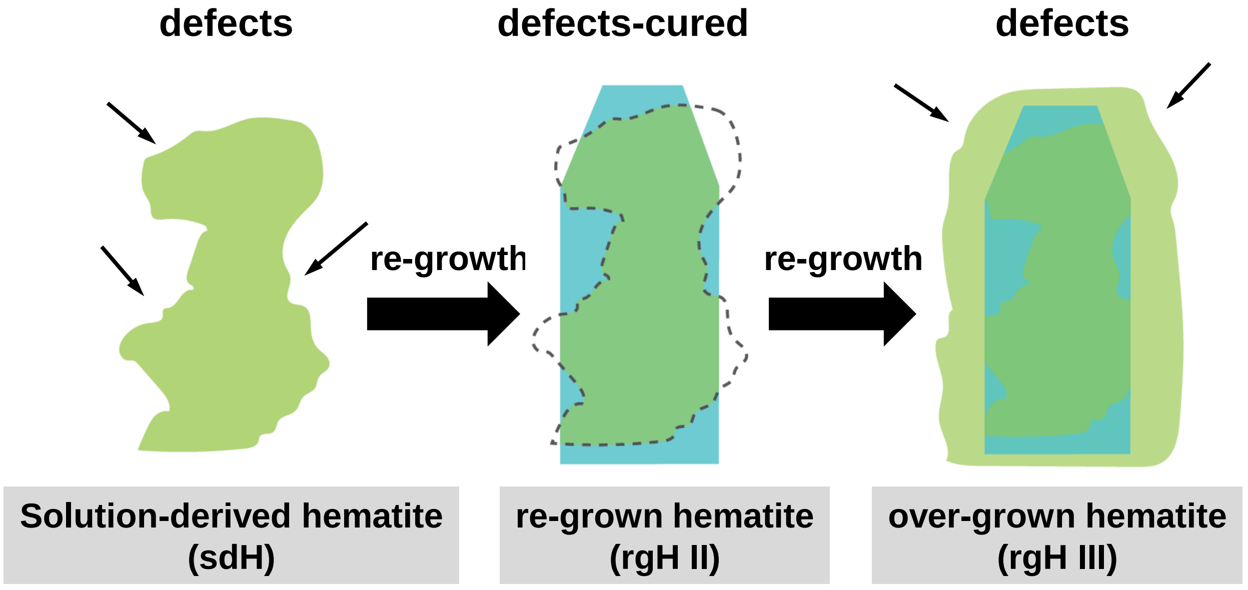

Supplementary Figure 1. Re-growth scheme of hematite.

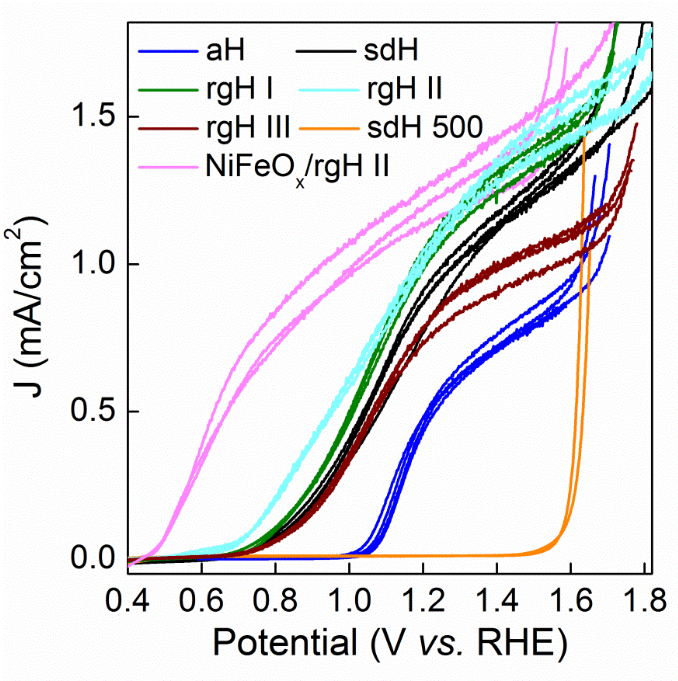

Supplementary Figure 2. Comparison of PEC performances of aH, sdH, rgH I, rgH II, rgH III and $NiFeO_x$/rgH II samples. The photocurrents of sdH 500 (annealed at 500°C) were less than 10μA. For clarity, dark currents were not shown.

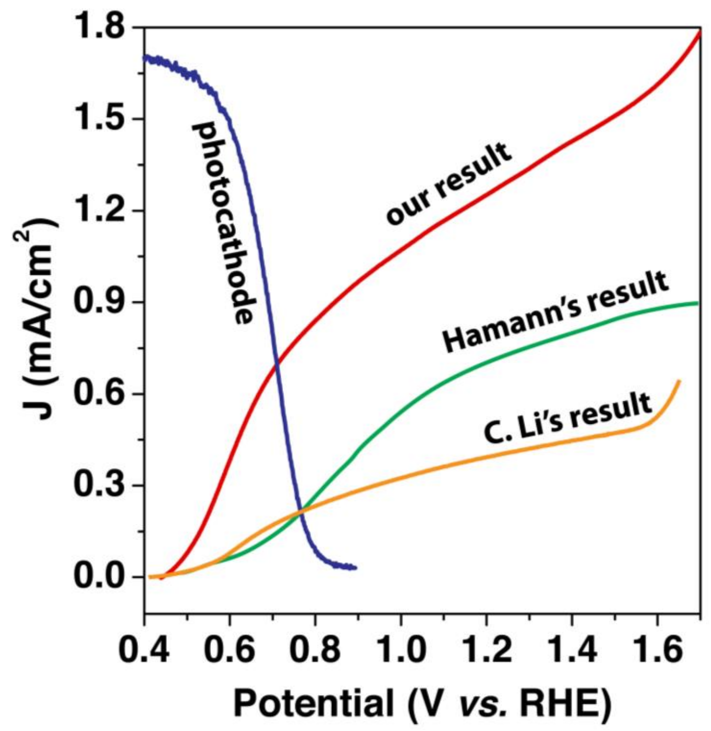

Supplementary Figure 3. Comparison of PEC performance of present result with those of Hamann and C. Li with a-Si photocathode[1,2]. The caveat of using simple figure-of-merit (such as V_{on}) to describe the performance of a photoelectrode is self-evident in the figure shown above. While the turn-on voltages may be comparable, the results obtained by Can Li et al.[1] and Thomas Hamann et al.[2] trail ours by a large margin in all other aspects.

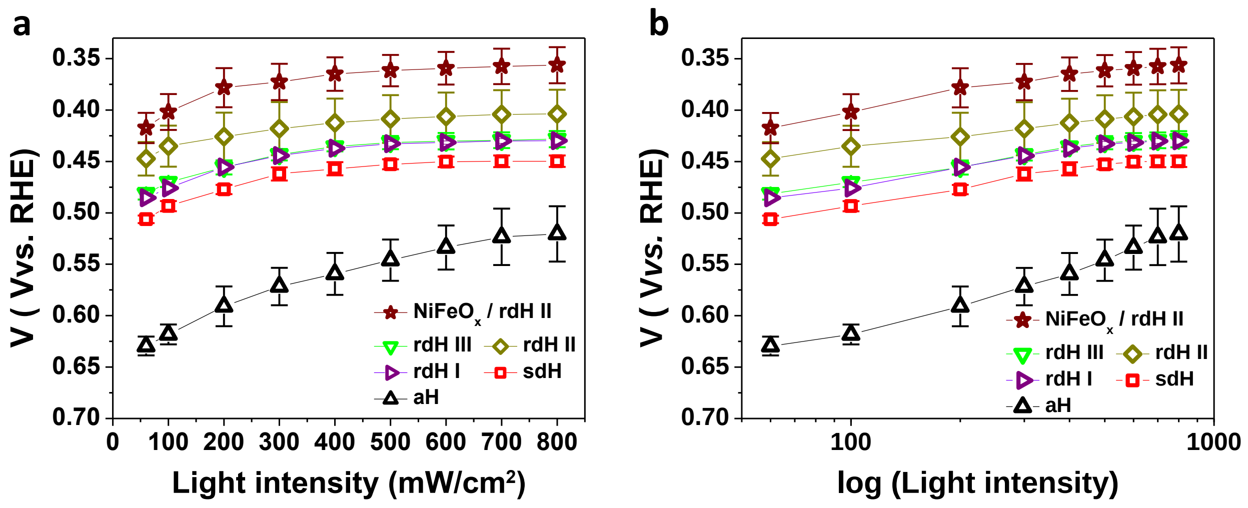

Supplementary Figure 4. Open circuit potential (OCP) of aH, sdH, rgH I, rgH II, rgH III and $NiFeO_x$ / rgH II measured with varying light intensity from 1-sun to 8-sun. The saturated OCPs report the true flatband potentials. (a) linear scale (b) Logarithm scale.

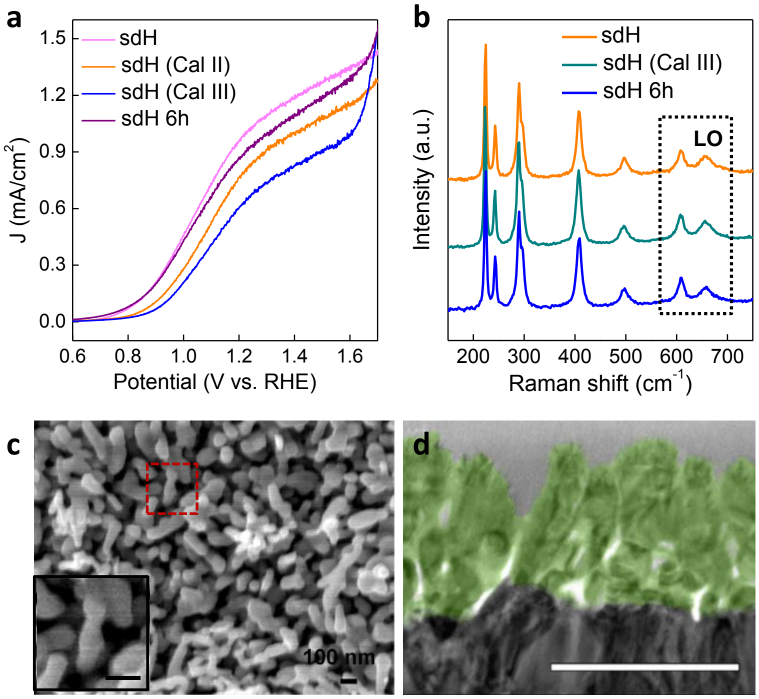

Supplementary Figure 5. Comparison of **(a)** PEC performance of hematite and **(b)** Longitudinal optical (LO) mode in Raman spectra upon repeated annealing process or prolonged reaction time. Here “Cal II” and “Cal III” represent samples that have been subjected to annealing conditions twice and three times, respectively. **(c)** Scanning electron micrographs; scale bar: 100 nm. and **(d)** transmission electron micrographs of cross-sectional sdH 6h samples prepared by FIB; scale bar: 500 nm.

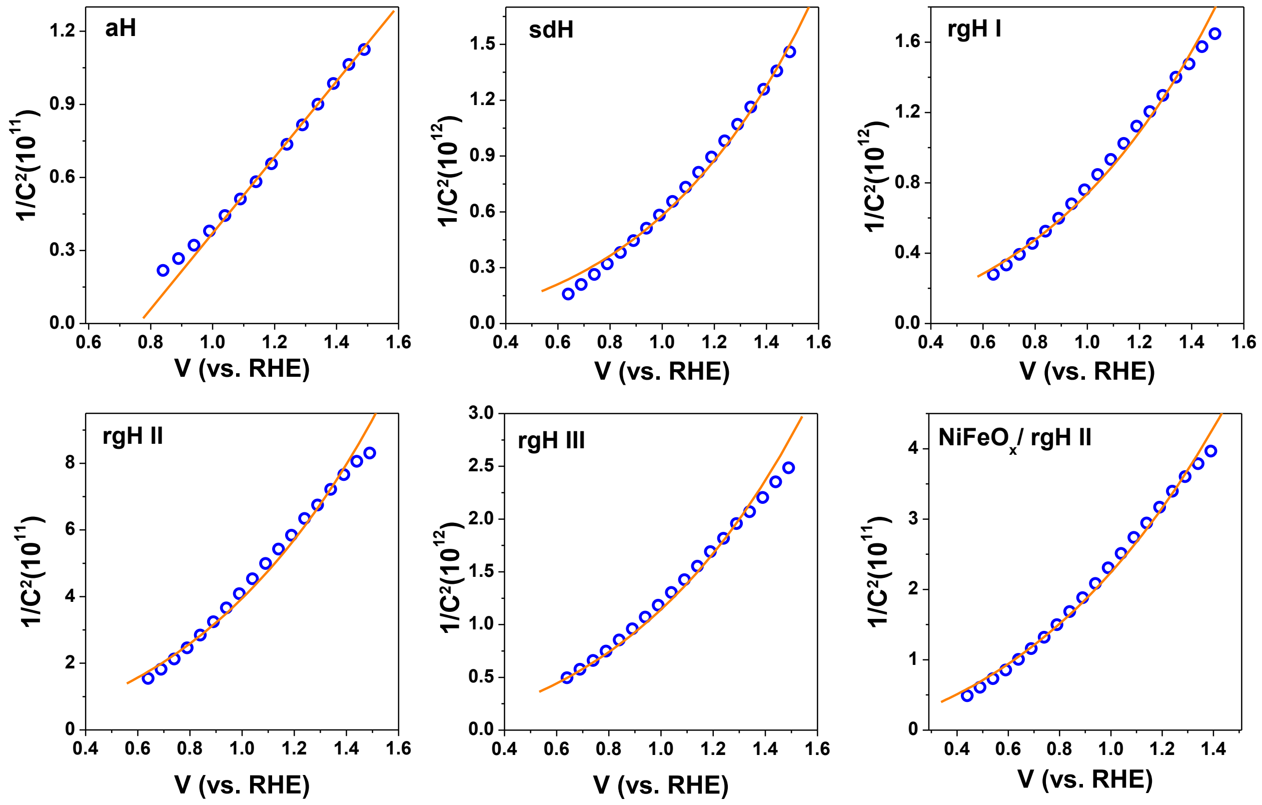

Supplementary Figure 6. Mott-Schottky plots of aH, sdH, rgH I, rgH II, rgH III and $NiFeO_x$ / rgH II in the dark condition. Amplitude=5mV, Potential range: 0.4~1.6V vs. RHE, Frequency: 1000Hz.

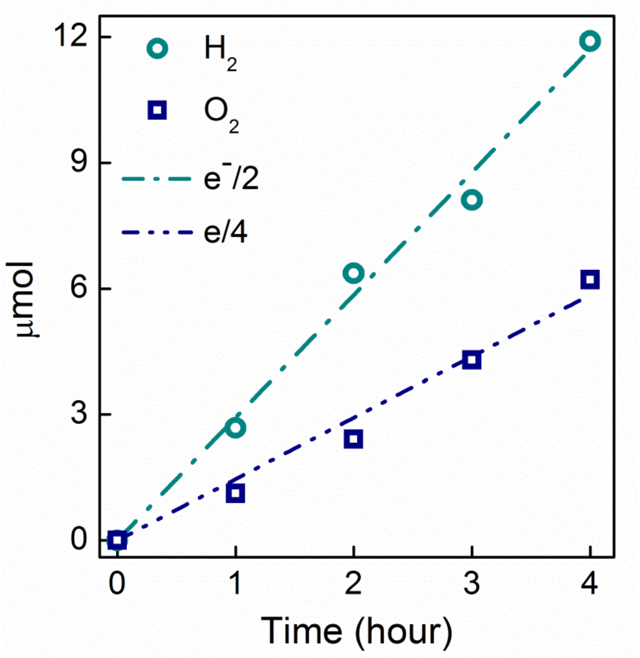

Supplementary Figure 7. Evolution of hydrogen/oxygen from the a-Si photocathode and $NiFeO_x$ modified rgH II. Faradaic efficiency approached to 100% ($e^-/2$ and $e^-/4$ denote the theoretical H_2 and O_2 amounts, respectively).

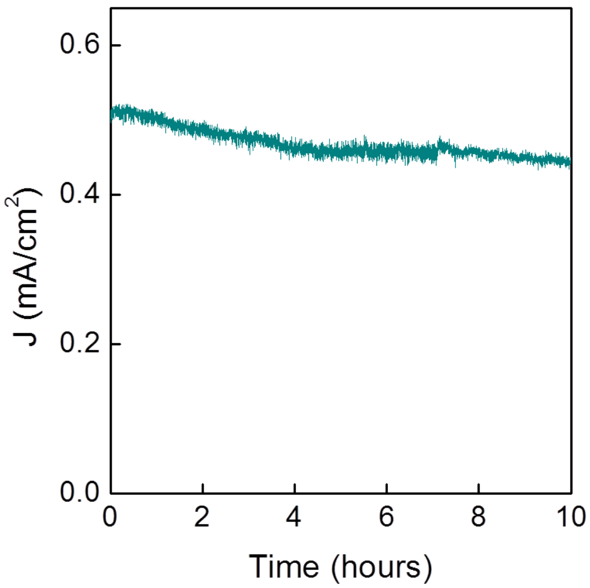

Supplementary Figure 8. Current densities of the $NiFeO_x$-decorated rgH II and a-Si in parallel configuration. The electrolyte was 0.5 *M* phosphate solution (pH 11.8).

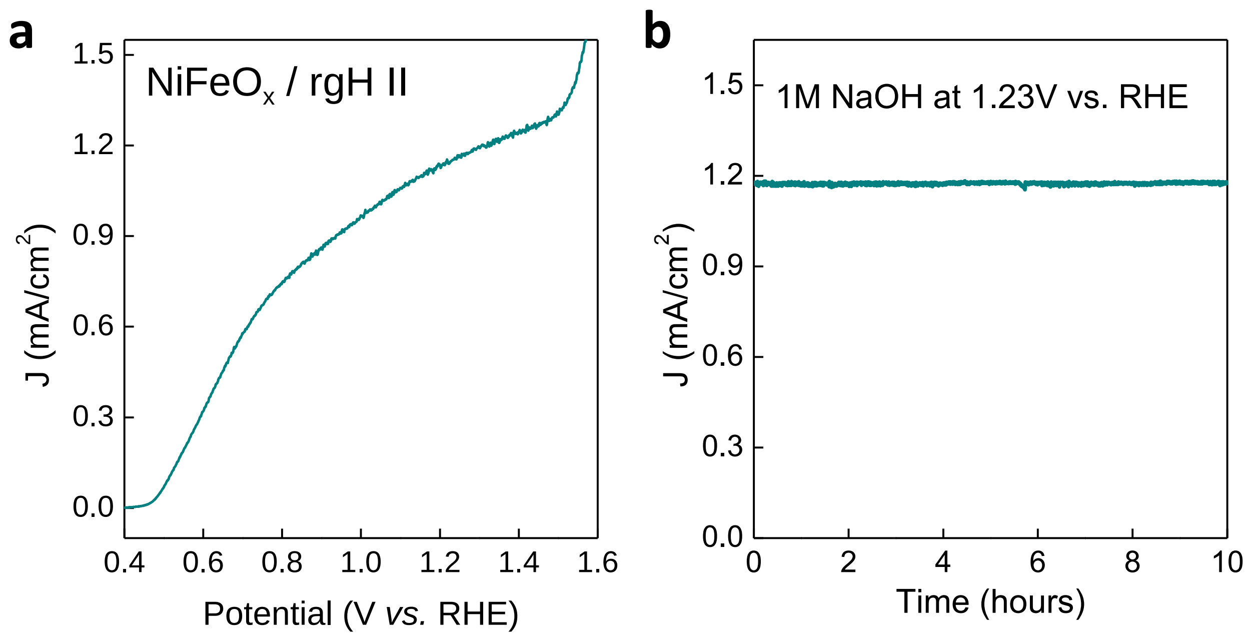

Supplementary Figure 9. (a) PEC behaviors of a NiFeO$_x$-decorated rgH II photoanode in 1 *M* NaOH. **(b)** Stability test during the 1st 10 h of operation in the same electrolyte solution at 1.23V *vs.* RHE. No obvious decrease in the photocurrent was observed.

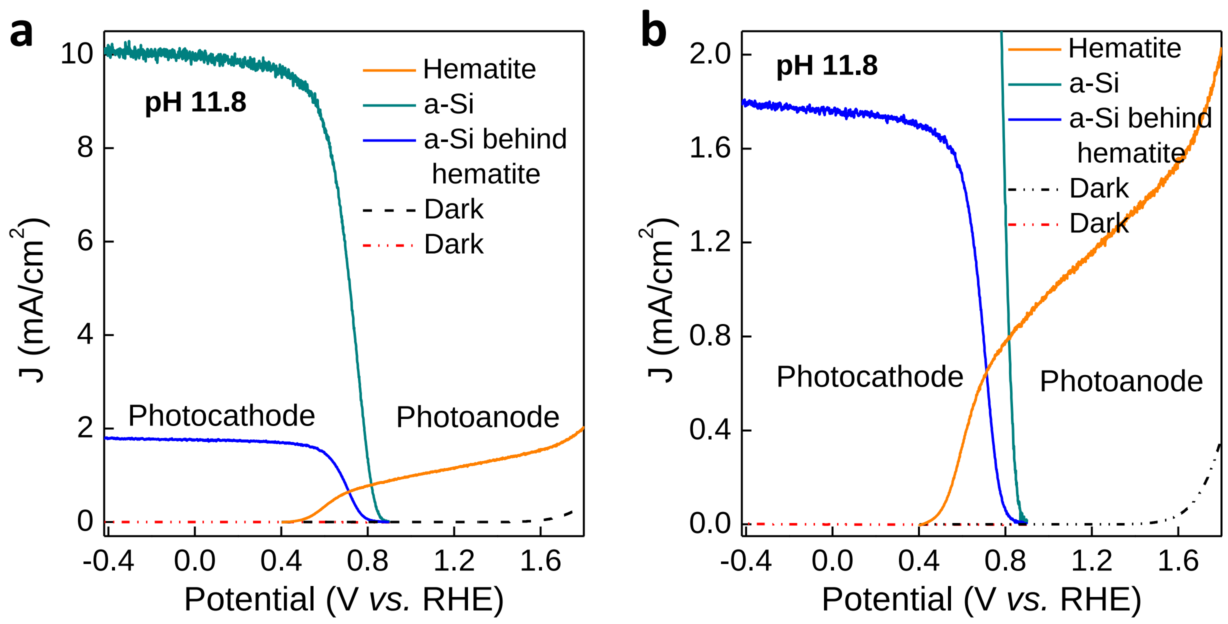

Supplementary Figure 10. (a) Current density-potential plots for a $NiFeO_x$-decorated rgH II and a-Si photocathode in 0.5 *M* phosphate solution (pH 11.8). **(b)** Magnified view of A. Due to the tandem cell configuration of this two-electrode system, light utilized by the a-Si photocathode was reduced, leading to a reduced photocurrent (from blue to green).

Supplementary Table 1. Comparision of (114)/(104) peak ratio and crystalline size of aH, aH 800, sdH, rgH I, rgH II, rgH III and sdH 6h.

	(104) peak intensity	***(110) peak intensity***	***(110)/(104) peak ratio***	***Size(nm)***
aH	149.2	95.3	0.64	19.0
aH 800	129.7	79.1	0.61	20.7
sdH	60.1	139.7	2.31	36.6
rgH I	137.0	900.9	6.57	37.1
rgH II	120.8	1214.2	10.1	41.9
rgH III	351.6	817	2.32	42.1
sdH 6h	207.1	474.8	2.29	37.4

Supplementary Table 2. Parameters (diameter, thickness and density of nanowire) and carrier concentrations of aH, sdH, rgH I, rgH II, rgH III, $NiFeO_x$/rgH II.

	Diameter (nm)	*Thickness (nm)*	D_{nw} *(cm^{-2})**	N_D *(cm^{-3})**
aH	-	20	-	1.26×10^{19}
sdH	72.2	130	1.8×10^{10}	2.55×10^{19}
rgH I	74.8	250	1.5×10^{10}	1.71×10^{19}
rgH II	77.4	460	1.0×10^{10}	2.81×10^{19}
rgH III	102.2	530	7.1×10^{9}	1.06×10^{19}
$NiFeO_x$/rgH II	77.4	460	1.0×10^{10}	4.12×10^{19}

Notes: * D_{nw}: *Density of nanowires per flat unit area (cm^2),* N_D*= Carrier concentration.*

Supplementary Table 3. Sumary of operation current and solar to hydrogen conversion efficiencies (STH) of $NiFeO_x$/rgH II and amorphous Si photocahode two-electrode water splitting system. Operation current was measured using both multimeter and potentionstat. The origin of the current increase is not entirely understood and requires further research. We speculate that the improved funtionalities of the catalyst may be an important reason.

	Photocurrent (mA/cm^2)	*STH (%)*
*Crossing point**	0.65	0.80
Initial Photocurrent	0.68	0.84
Stable Photocurrent	0.74	0.91

Notes: * *when photoanode & photocathode were measured separately*

Supplementary Discussion

The carrier concentrations were measured using Mott-Schottky plots, where the capacitance of the space charge region is plotted against the applied potential. Under ideal conditions, Mott-Schottky relation (1) describes the plots.

$$\frac{1}{C_s^2} = -\frac{N_D e}{2\varepsilon\varepsilon_0}\left[V_{sc} - V_{fb}\right] \quad \text{------- (1)}$$

Here C_s is the space charge layers capacitance per unit area, e is the fundamental charge of an electron, ε is the dielectric constant of the semiconductor, ε_0 is the permittivity of vacuum, N_D is the charge carrier density, V_{sc} is the applied potential, and V_{fb} is the flat band potential. By using this equation, N_D of aH was obtained (1.26×10^{19} cm^{-3}).

To calculate the N_D other hematite electrodes (sdH, rgH I, rgH II, rgH III and $NiFeO_x$/rgH II) we followed the calculation discussed previously by Mora-Seró *et al.* considering their nanoscale features[3].

Firstly, we assumed each nanowire hematite as cylinder of radius R and calculated their surface area by considering parameters such as electrode surface area, diameter, thickness, and density of nanowires per unit area and normalized the space charge capacitance to C_s (Supplementary Table 2). Their N_D were obtained by employing Eqs (2) and (3) derived from cylindrical shape instead of flat surface (Supplementary Table 2, Supplementary Figure 6).

$$V_{sc} = -\frac{N_D e}{2\varepsilon\varepsilon_0}\left[\frac{1}{2}\left(R^2 - x^2\right) + R^2 \ln\left(\frac{x}{R}\right)\right] \quad \text{------- (2)} \qquad C_s = \frac{2\varepsilon\varepsilon_0\, x^2}{R^2(R^2 - x^2)} \quad \text{-------- (3)}$$

Here, R is the radius of nanowire and x is the radius of quasineutral region from the center[3].

As shown in Supplementary Table 2, $\boldsymbol{N_D}$ of aH, sdH, rgH I, rgH II, rgH III and $NiFeO_x$/rgH II were1.26×10^{19}, 2.55 × 10^{19} , 1.71 × 10^{19}, 2.81 × 10^{19}, 1.06 × 10^{19} and 4.12 × 10^{19} cm^{-3}, respectively. The difference of the carrier density between aH and $NiFeO_x$-treated rgH II is approximately 3 times. It is far less than what is needed (~1000) to fully account for the Fermi level shift of 0.2 V.

Supplementary References

1. Han, J., Zong, X., Wang, Z. & Li, C. A hematite photoanode with gradient structure shows an unprecedentedly low onset potential for photoelectrochemical water oxidation. *Phys. Chem. Chem. Phys.* **16**, 23544-23548 (2014).
2. Zandi, O. & Hamann, T. W. Enhanced Water Splitting Efficiency Through Selective Surface State Removal. *J. Phys. Chem. Lett.* **5**, 1522-1526 (2014).
3. Mora-Seró, I., *et al.* Determination of Carrier Density of ZnO Nanowires by Electrochemical Techniques, *Appl. Phys. Lett.* **89**, 203117 (2006).
